# Probing the association between PD-1/PD-L1 expression and atrial fibrillation in peripheral blood of Han and Kazakh populations in Xinjiang region

**DOI:** 10.1097/MD.0000000000046309

**Published:** 2026-05-12

**Authors:** Wen Bai, Mierzhati Maimaiti, Xinwen Zhang, Xia Zhu, Muredili Saimi, Muhuyati Wulasihan

**Affiliations:** a Cardiovascular Center, Department of Cardiology, Urumqi Friendship Hospital, Urumqi, Xinjiang, China; b Science and Education Department, Urumqi Friendship Hospital, Urumqi, Xinjiang, China; c Department of Comprehensive Cardiology, The First Affiliated Hospital of Xinjiang Medical University, Urumqi, Xinjiang, China.

**Keywords:** atrial fibrillation, cytokines, immune cells, lymphocyte proliferation, PD-1/PD-L1

## Abstract

This study aimed to investigate the association between peripheral blood programmed cell death 1 (PD-1)/programmed death ligand 1 (PD-L1) expression and atrial fibrillation (AF) in the Han and Kazakh populations of Xinjiang. From May 2017 to December 2018, the Han and Kazakh groups in Xinjiang were selected for analysis using flow cytometry and other methodologies. Rigorous quality control measures were implemented to assess indicators associated with AF. Research has demonstrated that among the Han Kazakh group with AF, the positive expression rate of PD-1/PD-L1 on CD4+ T lymphocytes and dendritic cells in the AF cohort is lower than that observed in the control group, indicating a significant difference between the 2 ethnic groups. Additionally, the proliferation capacity of lymphocytes and the levels of cytokine secretion also exhibit differences that can be mitigated or reversed through treatment with interferon-α. Notably, the proliferation ability of the Han population is greater than that of the Kazakh population. Significant differences exist in the immune cell and cytokine profiles, as well as in PD-1/PD-L1 expression, between the Han and Kazakh groups with AF in Xinjiang and the control group. These differences may present potential targets for the treatment of AF.

## 1. Introduction

The pathogenesis of atrial fibrillation (AF), a prevalent and serious arrhythmia, is complex and not yet fully understood. In recent years, numerous studies have been conducted to elucidate the underlying causes of AF, particularly focusing on the role of inflammation in its development. Programmed death factor-1 (PD-1), a key member of the B7/CD28 family, plays a pivotal role in the human immune system. Comprising 288 amino acids, PD-1, in conjunction with its ligand PD-L, mediates negative regulatory signals during T-cell second signal transduction. This process inhibits the expansion and function of T effector cells, subsequently inducing immune tolerance.^[1,2]^ This mechanism has been applied in the context of tumor immune tolerance. Additionally, PD-1 has been observed to induce the immunomodulatory roles of specific subpopulations of natural killer T-cells, B cells, monocytes, and dendritic cells (DCs) following activation. Furthermore, the ligands of PD-1, programmed death ligand 1 (PD-L1) and PD-L2, exhibit distinct expression patterns. PD-L1 is expressed at low levels in antigen-presenting cells (APCs), with expression increasing rapidly following the activation of T-cells and APCs. It is also expressed in various non-hematopoietic cell types. In contrast, PD-L2 is expressed at high levels primarily in DCs and macrophages. Notably, PD-L1 is expressed at low levels on APCs, which increases rapidly after the activation of T-cells and APCs, while PD-L2 is predominantly expressed at high levels on DCs and macrophages.^[3,4]^ The cell-specific and tissue-specific distribution of PD-1 ligands enables PD-1 to mediate distinct stages of T-cell activation by binding to various ligands.^[4,5]^ An increasing number of studies have demonstrated that T lymphocyte activation is a significant pathogenic factor in the development of AF, with inflammation emerging as a pivotal element in its pathogenesis.^[5]^ Serum levels of inflammatory biomarkers are elevated in individuals with AF. Furthermore, inflammatory markers are highly expressed in the myocardial tissues of patients with AF and in animal models of the condition. Additionally, anti-inflammatory drugs have shown efficacy in managing cases of AF. It is evident that inflammatory responses are closely associated with several pathological processes in AF, including oxidative stress, apoptosis, and fibrosis. Collectively, these processes contribute to the formation of the AF maintenance matrix. Moreover, inflammation is linked to endothelial dysfunction, platelet activation, and the activation of the coagulation cascade, which subsequently increases the risk of thrombosis. In-house research has demonstrated that PD-1/PD-L1 is involved in the regulation of inflammation in AF.^[6,7]^

A substantial body of evidence from numerous studies has confirmed that the proliferative capacity of T lymphocytes and the detection of inflammatory factors serve as effective indicators of the inflammatory state within the body.^[8,9]^ Several biomarkers, including C-reactive protein (CRP), interleukin-6 (IL-6), interleukin-10 (IL-10), and NLR, have been identified as closely associated with the occurrence and prognosis of AF as well as adverse cardiovascular events.^[10–12]^ Any cause of inflammatory response in the body may increase the risk of AF development. For instance, conditions such as nonalcoholic fatty liver disease and metabolic syndrome further suggest that inflammation is a significant predictor of the microenvironment in which AF is sustained.^[13]^ In the context of inflammatory regulation in AF, low expression of PD-1 on predominantly CD4+ T cells has been observed to promote T-lymphocyte activation and increase the inflammatory load in vivo. This phenomenon has been linked to the development of cardiomyocyte fibrosis and structural remodeling. Notably, the involvement of CD8+ T cells does not appear to be a pivotal factor in the hyperinflammatory state of AF in vivo. Conversely, PD-L1 on DCs undergoes a subsequent isotropic change.^[14,15]^ To verify this observation, the present study employed a mixed lymphocyte reaction assay to ascertain the capacity for T lymphocyte activation and the expression levels of TH1/TH2-like cytokines in the coculture fluid, with the aim of analyzing the characteristics of their immunomodulation. The results demonstrated that the blocking effect of PD-L1 site inhibitors was more pronounced than that of PD-1 site blockers. Furthermore, the anti-PD-L1 monoclonal antibody did not induce de novo inhibitory signaling in T cells, as it did not bind to PD-1. This suggests that the anti-PD-L1 monoclonal antibody may have greater efficacy than anti-PD-1 antibodies. However, evidence also indicates that PD-L1 is widely expressed in normal tissues, which suggests that the use of anti-PD-L1 monoclonal antibodies may result in a higher incidence of immune-related toxicity. For this reason, we elected to upregulate PD-L1 expression on DCs to determine whether it could mitigate the inflammatory burden in the AF population. It is anticipated that this study will provide a crucial foundation for a comprehensive understanding of the etiology of AF, particularly regarding the immunoregulatory mechanisms and the role of inflammation in AF across diverse ethnic populations. Furthermore, it is hoped that the findings will pave the way for novel approaches to the diagnosis and treatment of AF.

## 2. Materials and methods

### 2.1. Research object

Between May 2017 and December 2018, the population attending the Department of Comprehensive Cardiology at the First Affiliated Hospital of Xinjiang Medical University was selected as the screening subjects. Ultimately, 200 cases each from the Han and Kazakh populations, who were permanently residing in the Xinjiang region, were chosen for the study. The experimental group comprised 100 patients with a definitive diagnosis of AF, while the control group included 100 individuals with no history of AF.

### 2.2. Case group inclusion and exclusion criteria

Inclusion criteria: The study population was selected based on the criteria outlined in the ACC and AHA 2006 Guidelines for the Treatment of AF, requiring a documented history of AF and a confirmed diagnosis based on either a 24-hour ambulatory electrocardiogram or a 12-lead electrocardiogram.

Exclusion criteria: All study participants must be excluded if they have any of the following conditions: cardiac valvular disease, congenital heart disease, rheumatoid immune disease, severe cardiac, hepatic, or renal disease, recent trauma or surgical treatment, recent infections (fever > 37.4°C), malignancy, immune system disorders, hyperthyroidism, or if they are receiving anti-inflammatory or immunological treatments.

### 2.3. Control group inclusion and exclusion criteria

Inclusion criteria: Non-AF patients with no history of arrhythmia or risk factors, as determined by routine clinical examination.

Exclusion criteria: All study participants must be excluded if they have any of the following conditions: cardiac valvular disease, congenital heart disease, rheumatoid immune disease, severe cardiac, hepatic, or renal disease, recent trauma or surgical treatment, recent infections (fever > 37.4°C), malignancy, immune system disorders, hyperthyroidism, or if they are receiving anti-inflammatory or immunological treatments.

All patients were thoroughly informed and provided written consent for their participation in this study, which received approval from the Ethics Committee of the First Affiliated Hospital of Xinjiang Medical University (approval number: 20150225-80).

## 3. Content and methodology

### 3.1. Experimental materials

Primary reagent: The following reagents were utilized in this study: mouse antihuman CD4-FITC, mouse antihuman CD8-PE, mouse antihuman CD3-CY5.5, mouse antihuman CD11c-APC, mouse antihuman CD123-APC, mouse antihuman CD279-PE-cy7, and murine antihuman PD-L1-FITC, all purchased from Pharmingen, USA. Additionally, RPMI 1640 medium, erythrocyte lysate, and BD buffer were sourced from Hyclone, China, and Solebo, China, respectively. Poly IC solution was obtained from Biosharp, China, while phosphate-buffered saline solution was also acquired from Hyclone, China. Lymphocyte isolation solution and sheath fluid were purchased from BD, USA. Furthermore, fetal bovine serum was obtained from 4 Seasons, and dimethyl sulfoxide was acquired from Sigma, USA. Lastly, a human cytokine ELISA analysis kit was sourced from BD, USA.

Major instrumentation: The study utilized several key instruments, including a flow cytometer from Beckman Coulter (Brea), a 37-degree Celsius thermostat from Thermo Fisher Scientific (Waltham), a carbon dioxide (CO_2_) cell culture incubator also from Thermo Fisher Scientific, an inverted microscope from Nikon (Japan), an ultraclean bench from Jinan Kangbao (China), and an ultracentrifuge from Thermo Fisher Scientific.

### 3.2. Primary solution and preparation

Preparation of the Dulbecco’s modified Eagle medium (DMEM) cell culture solution involves combining 100 mL of DMEM culture solution with 3.7 g of sodium bicarbonate and 0.2 g of L-glutamine in 1000 mL of ultrapure water. A double antibiotic solution is then added to achieve a concentration of 100 U/mL for both penicillin and streptomycin. Following this, 10% fetal bovine serum is incorporated into the mixture. The solution is filtered and sterilized using a sterile 0.22 µm membrane, dispensed into sterile 50 mL centrifuge tubes, and stored in a refrigerator at 4°C. The sample is maintained at this temperature.

## 4. Experimental steps

### 4.1. Isolation of single nucleated cells from peripheral blood

In accordance with the experimental methodology outlined in the instruction manual for the Peripheral Blood Single Nucleated Cell Isolation Solution, the white membrane layer present in the mixture was extracted and identified as the lymphocyte layer. Subsequently, the cells were resuspended in the prepared DMEM culture medium for storage.

### 4.2. Flow cytometry isolation of CD4+ T lymphocytes, CD8+ T lymphocytes and DC cells

The single nucleated cells, separated using a lymphocyte isolation solution, were allocated into 3 tubes (A, B, and C), to which fluorescent monoclonal antibodies were added. Tube A received 20 µL of CD4-FITC antibody, tube B received 20 µL of CD8-PE antibody, and tube C received 20 µL of CD11chighCD123low antibody. The tubes were subsequently incubated for 15 minutes in the absence of direct light. Following this incubation period, the cells were sorted using flow cytometry, and the purity of the sorted cells was assessed. After sorting, the cells were inoculated into 6-well plates, with 2 mL of DMEM cell culture medium added to each well, and subsequently cultured in a cell culture incubator.

### 4.3. Culture of DC cells

Following sorting, DCs are readily differentiated into macrophages and consequently lose their antigen-presenting function in vitro. To inhibit this differentiation and retain their antigen-presenting capabilities, 50 μg/mL of poly I:C was added to the DC culture medium. Simultaneously, an equal quantity of purified DCs was isolated, and 1000 IU/mL of interferon-α (IFN-α) was introduced to upregulate the expression of PD-L1 on the DCs. The expression level of PD-L1 was subsequently measured by flow cytometry after a 24-hour incubation period.

### 4.4. Mixed lymphocyte reaction (MLR) assay

Immune cells were isolated using flow cytometry to determine their cell count and subsequently suspended in DMEM culture medium to calculate the cell concentration. In each experimental group, purified DC and CD4+/CD8+ T cells were added in varying ratios. The CD4+ and CD8+ T cells were sourced from the peripheral blood of allogeneic healthy individuals, with their ratios set at 1:5, 1:10, 1:20, and 1:40. The cell mixture was cultured in 96-well plates, each containing a total of 10,000 cells and a volume of 200 μL. The plates were incubated in a 5% CO₂ environment at 37°C for 5 days. Following this incubation, 20 μL of cell counting kit-8 solution was added to each well to assess cell concentration. The plates were maintained at 37°C with 5% CO₂ throughout the experiment. After the 5-day incubation period, absorbance at 450 nm was measured over a 6-hour timeframe, concurrently plotting a standard curve for cell proliferation detection. Additionally, the culture supernatant was collected for subsequent cytokine analysis.

### 4.5. ELISA kits for the determination of cytokines in coculture supernatants

In accordance with the methodology outlined in the ELISA kit, the standards are established to generate the standard curve. Subsequently, the samples and each working solution are added following the prescribed steps. Once the reaction is complete and thoroughly mixed, the OD450 (optical density) value is measured promptly (within 3 minutes) in the enzyme lab. The data is then saved in the instrument, and a hard copy of the results is produced.

### 4.6. Detection of PD-1 expression levels on human peripheral blood CD4+/CD8+ T cells by flow cytometry

Flow cytometry was employed to assess the expression levels of PD-1 on CD4+ and CD8+ T cells in human peripheral blood. A 400 μL aliquot of the isolated peripheral blood mononuclear cell (PBMC) suspension was added to flow cytometry tubes, with each tube containing more than 1 × 10⁵ cells. Fluorescently labeled antibodies were added as follows: in Tube A (for detection of PD-1 on CD4+ T cells), 20 μL each of CD3-CY5.5, CD4-FITC, and PD-1-PE were added; in Tube B (for detection of PD-1 on CD8+ T cells), 20 μL each of CD3-CY5.5, CD8-FITC, and PD-1-PE were added. In parallel, blank controls (without antibodies) and isotype controls (using irrelevant antibodies of the same subtype as the primary antibodies) were prepared. The samples were incubated in the dark for 15 minutes before acquisition on the flow cytometer. Data analysis was performed using CellQuest software.

The following gating strategy was applied to identify the target cell populations and calculate the PD-1 positive rate:

① First, the PBMC population was gated based on forward scatter and side scatter to exclude cellular debris and nonviable cells;

② Second, total T cells were identified as CD3+ cells within the PBMC gate;

③ Third, CD4+ T cells and CD8+ T cells were distinguished based on CD4-FITC and CD8-FITC fluorescence signals, respectively;

④ Finally, within the CD4+ and CD8+ T-cell populations, the threshold for PD-1-PE positivity was determined based on the fluorescence signal from the isotype control. The percentage of PD-1-positive cells within each T-cell subset was then calculated, representing the PD-1 expression rate.

### 4.7. Detection of PD-L1 expression level on human peripheral blood DC cells by flow cytometry

Flow cytometry was employed to assess the expression levels of PD-L1 on DCs in human peripheral blood. A 400 μL aliquot of the isolated PBMC suspension was added to flow cytometry tubes, with each tube containing more than 1 × 10⁵ cells. For each sample, blank controls (without antibodies) and isotype controls (using irrelevant antibodies of the same subtype as the primary antibodies) were prepared. Subsequently, fluorescently labeled antibodies were added as follows: 5 μL of CD11c-APC, 20 μL of CD123-PE, and 20 μL of PD-L1-FITC. The samples were incubated in the dark for 15 minutes before acquisition on the flow cytometer. Data analysis was performed using CellQuest software.

The following gating strategy was applied to identify the target cell populations and calculate the PD-L1 positive rate:

① First, the PBMC population was gated based on forward scatter and side scatter to exclude cellular debris and nonviable mononuclear cells;

② Second, within the PBMC gate, myeloid DCs were identified as CD11c^high CD123^low double-positive cells, consistent with the sorting criteria used for DC populations to ensure uniformity in cell definition;

③ Finally, within the myeloid DC population, the threshold for PD-L1-FITC positivity was determined based on the fluorescence signal from the isotype control. The percentage of PD-L1-positive cells within the myeloid DC subset was then calculated, representing the PD-L1 expression rate.

### 4.8. Quality control

Cell isolation and culture are performed by laboratory professionals who have received the necessary training; flow cytometry was carried out by a third-party professional organization; and this phase of the experiment employed a single-blind method, ensuring that the analysts responsible for testing and analyzing the results were unaware of the sample groupings.

### 4.9. Data analysis and statistics

Data were collected and organized using Excel 2023 software, with subsequent analyses conducted using both SPSS 26.0 (IBM SPSS Statistics 26.0, Armonk) and GraphPad Prism software. For normally distributed data, the mean ± standard deviation ($\bar{x}\pm s$) was utilized for group comparisons; for non-normally distributed data, the median (*M*) along with the upper and lower interquartile ranges (Q25, Q75) were employed for comparisons between groups. In these instances, the rank-sum test was applied. For count data, case rates or composition ratios were reported, and group comparisons were performed using either the χ² test or Fisher’s exact test. A *P*-value of <.05 was considered statistically significant.

## 5. Results

### 5.1. Comparison of general clinical data between the 2 groups

In this study, we selected a total of 400 individuals from the Han and Kazakh populations in the Xinjiang region who met the inclusion criteria. These individuals were divided into 4 groups: a Han AF group (100 cases), a Han control group (100 cases), a Kazakh AF group (100 cases), and a Kazakh control group (100 cases). Additionally, a PD1 group consisting of 100 cases was included. The history of AF, both with and without the condition, was considered in the formation of these groups. DNA sequencing was conducted on the PD1 group, specifically identifying the PD1.1 and PD1.5 gene loci of the selected PD-1 genes through a review of the relevant literature. Prior to sequencing, data were collected uniformly by the research team, which included the subject’s name, age, gender, ethnicity, smoking history, comorbidities (coronary artery disease, hypertension, diabetes mellitus), medication history (angiotensin-converting enzyme inhibitor [ACEI]/angiotensin II receptor blocker [ARB], beta-blockers, calcium antagonists, statins), and biochemical indices (triglycerides, total cholesterol, white blood cell count, lymphocyte count). Furthermore, a 12-lead electrocardiogram and a 24-hour ambulatory electrocardiogram were performed, along with an ultrasound examination of the left atrial internal diameter (LID). The medical history was also assessed, including any previous cases of valvular heart disease, congenital heart disease, intermarriage or close blood relations, severe heart, liver, or kidney disease, recent trauma or surgical treatment, rheumatological and immunological diseases, and any recent infections (fever > 37°C). Additionally, the following conditions were excluded: malignancy, history of hematological disorders, and immune system disorders. A training program was implemented for data collectors, and a uniform design of forms was established to ensure the accuracy of data collection.

A comparison of the general baseline data within the same ethnic group of the population included in the study revealed no statistically significant differences in age, sex, smoking history, comorbidities (coronary artery disease, hypertension, diabetes mellitus), medication history (ARB/ACEI, β-blockers, calcium channel blocker [CCB]), triglyceride levels, total cholesterol, and leukocyte counts between the AF group and the control group (*P* > .05). No statistically significant differences were observed between the AF and control groups in terms of age, gender, smoking history, comorbidities (coronary artery disease, hypertension, diabetes), medication history (β-blockers, CCB), triglycerides, total cholesterol, and lymphocyte counts (*P* > .05). This suggests that the 2 groups were adequately matched. However, within the Han Chinese population, there was a notable discrepancy in the medication history between the AF group and the control group (*P* < .05), with statins being utilized more frequently in patients with AF. Additionally, significant differences in lymphocyte counts were observed between the 2 groups (*P* < .05), with patients with AF exhibiting higher lymphocyte levels than those in the control group. Furthermore, the LID differed significantly between the 2 groups (*P* < .05), with patients with AF showing a greater LID compared to the control group. A significant difference was observed in the history of ACEI/ARB use between the AF group and the control group (*P* < .05), with patients in the AF group utilizing ACEI/ARB more frequently. Similarly, a difference was noted in the history of statin use between the AF group and the control group (*P* < .05), with patients with AF using statins more often. Patients with AF exhibited a greater propensity to utilize statins, and a notable disparity was observed in white blood cell counts between the 2 groups (*P* < .05), with patients with AF displaying elevated lymphocyte levels. Additionally, a statistically significant difference was evident in LID between the 2 groups (*P* < .05), with the LID of patients with AF being markedly larger than that of the control group (Table 1).

**Table 1:** Comparison of general information between the AF and control groups in the Han and Kazakh populations.

Variable	Kazakh		*P*	Han		*P*
	AF (100)	Control (100)		AF (100)	Control (100)	
Age (mean ± SD, yr)	59.76 ± 12.50	57.59 ± 12.04	.555	69.79 ± 10.61	65.45 ± 10.89	.670
Sex (male/female, %)	70/30	76/24	.426	56/44	52/48	.370
Smoking (%, n)	66	62	.659	72	71	1.000
Complications						
Coronary heart disease (%, n)	14	24	.212	38	29	.272
Hypertension (%, n)	34	32	.881	26	22	.620
Diabetes (%, n)	90	78	.330	84	74	.118
Drug history						
ARB/ACEI (%, n)	78	52	.000	52	52	1.000
βRB (%, n)	68	62	.459	56	54	.887
CCB (%, n)	76	66	.160	68	56	.109
Statins (%, n)	80	48	.000	68	46	.003
Triglycerides (mmol/L, mean ± SD)	1.15 ± 0.69	1.47 ± 1.00	.323	1.48 ± 1.07	1.57 ± 0.83	.460
Cholesterol (mmol/L, mean ± SD)	3.78 ± 1.39	3.72 ± 0.92	.103	3.76 ± 1.18	3.70 ± 1.00	.271
Leukocyte count (×10^9^/L, mean ± SD)	6.89 ± 1.53	6.39 ± 1.38	.042	5.74 ± 1.57	6.13 ± 1.38	.566
Lymphocyte count (×10^9^/L, mean ± SD)	2.16 ± 0.80	2.04 ± 0.65	.270	1.88 ± 0.68	1.80 ± 0.55	.039
Left atrial inner diameter (mm, mean ± SD)	40.30 ± 6.70	35.30 ± 5.18	.008	38.70 ± 6.33	34.74 ± 4.39	.000

ACEI = angiotensin-converting enzyme inhibitor, AF = atrial fibrillation, ARB = angiotensin II receptor blocker, CCB = calcium channel blocker, SD = standard deviation.

A comparison of baseline characteristics between the AF group and the control group from the Xinjiang region, encompassing various ethnicities, was conducted. In the AF group, no significant differences (*P* > .05) were observed between the Han and Kazakh nationalities regarding gender, smoking history, complications (such as hypertension and diabetes), medication history (including β-blockers, CCBs, and statins), total cholesterol levels, and left atrial diameter. However, a significant difference in age was noted between the 2 groups in the AF cohort (*P* < .05), with the Han AF population being older than their Kazakh counterparts. Additionally, a significant difference in the prevalence of coronary heart disease complications was identified, with a higher incidence among Han individuals (*P* < .05). Furthermore, there was a notable difference in ACEI/ARB medication history, with a greater proportion of Kazakh AF patients receiving statin therapy (*P* < .05). There is a significant difference in triglyceride levels between the 2 groups (*P* < .05) are noted between Han and Kazakh populations regarding smoking history, complications (such as coronary heart disease, hypertension, and diabetes), medication history (including ACEI/ARB, β-blockers, CCB, and statins), triglyceride levels, total cholesterol, white blood cell count, and left atrial diameter. However, a significant difference in age is observed between the 2 groups in the control population (*P* < .05), with the Han non-AF population being older than the Kazakh population. Furthermore, there is a notable difference in gender distribution between the 2 ethnic groups (*P* < .05), as the proportion of males is higher in the Kazakh non-AF population. Lastly, there is a significant difference in lymphocyte count between the 2 ethnic groups (*P* < .05), with Han individuals exhibiting higher lymphocyte levels than those of Kazakh ethnicity (Table 2).

**Table 2:** Comparison of general information between Han Chinese and Kazakh populations in the AF and control groups.

Variable	AF		*P*	Control		*P*
	Han (100)	Kazakh (100)		Han (100)	Kazakh (100)	
Age (mean ± SD, yr)	69.79 ± 10.61	59.76 ± 12.50	.000	65.45 ± 10.89	57.59 ± 12.04	.000
Sex (male/female, %)	56/44	70/30	.057	52/48	76/24	.001
Smoking (%, n)	72	66	.445	71	62	.176
Complications						
Coronary heart disease (%, n)	38	14	.000	29	24	.522
Hypertension (%, n)	26	34	.280	22	32	.151
Diabetes (%, n)	84	90	.293	74	78	.620
Drug history						
ARB/ACEI (%, n)	52	78	.000	52	52	1.000
βRB (%, n)	56	68	.109	54	62	.316
CCB (%, n)	68	76	.270	56	66	.192
Statins (%, n)	68	80	.076	46	48	.887
Triglycerides (mmol/L, mean ± SD)	1.48 ± 1.07	1.15 ± 0.69	.011	1.57 ± 0.83	1.47 ± 1.00	.472
Cholesterol (mmol/L, mean ± SD)	3.76 ± 1.18	3.78 ± 1.39	.940	3.70 ± 1.00	3.72 ± 0.92	.859
Leukocyte count (×10^9^/L, mean ± SD)	5.74 ± 1.57	6.89 ± 1.53	.000	6.13 ± 1.38	6.39 ± 1.38	.194
Lymphocyte count (×10^9^/L, mean ± SD)	1.88 ± 0.68	2.16 ± 0.80	.007	1.80 ± 0.55	2.04 ± 0.65	.005
Left atrial inner diameter (mm, mean ± SD)	38.70 ± 6.33	40.30 ± 6.70	.084	34.74 ± 4.39	35.30 ± 5.18	.411

ACEI = angiotensin-converting enzyme inhibitor, AF = atrial fibrillation, ARB = angiotensin II receptor blocker, CCB = calcium channel blocker, SD = standard deviation.

### 5.2. PD-1 expression on peripheral blood CD4+ cells in Han and Kazakh patients with AF

**Figure 1.**
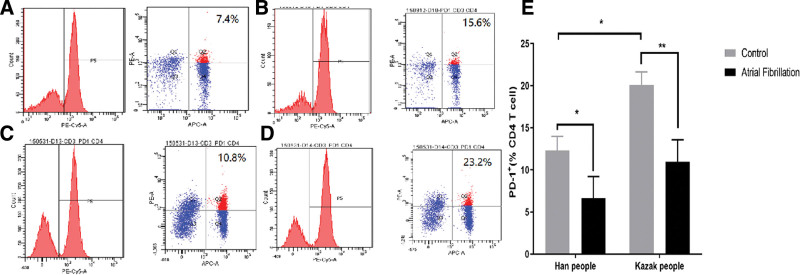
Expression of PD-1 on the surface of peripheral blood CD4+ T lymphocytes in the AF group and the non-AF group. (A) Han AF; (B) Han non-AF; (C) Kazakhs AF; (D) Kazakhs non-AF; and (E) PD-1 versus PD-L1 is expressed on the surface of the cells; ^*^P < .05, ^**^P < .01. AF = atrial fibrillation, PD-1 = programmed cell death 1, PD-L1 = programmed death ligand 1.

In the Han population, the positive expression rate of PD-1 on CD4+ T lymphocytes was (7.21 ± 0.76)% in the AF group and (13.24 ± 0.42)% in the control group, with a statistically significant difference observed between the 2 groups (*P* < .05). This indicates that the expression of PD-1 on CD4+ T lymphocytes in the Han Chinese population with AF was higher than that in the control group (Fig. 1A, B).

In the Kazakh population, the positive expression rate of PD-1 on CD4+ T lymphocytes was (10.1 ± 0.76)% in the AF group and (21.24 ± 0.42)% in the control group, also showing a statistically significant difference (*P* < .05). This suggests that the expression of PD-1 on CD4+ T lymphocytes in the Kazakh AF population was higher than that in the control group (Fig. 1C, D).

The results of flow cytometry demonstrated that the expression level of PD-1 on the surface of CD4+ T lymphocytes was significantly lower in both the Han and Kazakh AF groups compared to the non-AF group, with statistically significant results (*P* < .05, *P* < .01). Further analysis indicated that the expression level of PD-1 on CD4+ T cells was higher in the Kazakh group than in the Han Chinese group (Fig. 1E).

### 5.3. PD-1 expression on peripheral blood CD8+ cells in Han and Kazakh patients with AF

**Figure 2.**
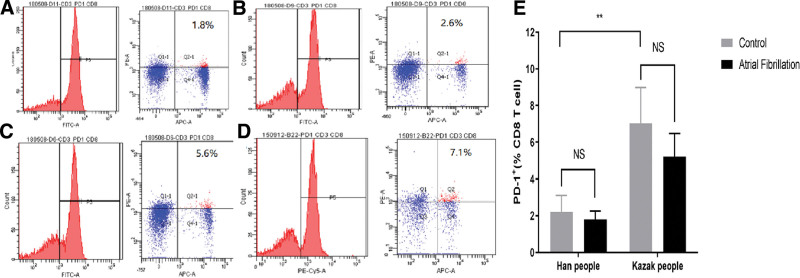
Expression of PD-1 on the surface of peripheral blood CD8+ T lymphocytes in the AF and non-AF groups. (A) Han AF; (B) Han non-AF; (C) Kazakhs AF; (D) Kazakhs non-AF; and (E) PD-1 versus PD-L1 is expressed on the surface of the cells; ^*^P < .05, ^**^P < .01. AF = atrial fibrillation, PD-1 = programmed cell death 1, PD-L1 = programmed death ligand 1.

In the Han population, the positive expression rates of PD-1 on CD8+ T lymphocytes in the AF group and the control group were (1.9 ± 0.46%) and (2.2 ± 0.42%), respectively. There was no statistically significant difference between the 2 groups (*P* > .05), indicating that the expression of PD-1 on CD8+ T lymphocytes in the Han Chinese AF population did not differ significantly from that in the control group (Fig. 2A, B).

In the Kazakh population, the positive expression rates of PD-1 on CD8+ T lymphocytes in the AF group and the control group were (5.3 ± 0.46%) and (6.8 ± 0.42%), respectively, with no statistically significant difference observed between the 2 groups (*P* > .05). Similarly, there was no significant difference in the expression of PD-1 on CD8+ T lymphocytes between the Kazakh population with AF and the control group (Fig. 2C, D).

The results of flow cytometry demonstrated that there was no statistically significant difference in the expression of PD-1 on CD8+ T lymphocytes between the Han and Kazakh AF groups compared to the non-AF group (*P* > .05). However, the expression level of PD-1 on CD8+ T cells was found to be higher in the Kazakh group than in the Han population group (Fig. 2E).

### 5.4. PD-L1 expression on peripheral blood DC cells of Han and Kazakh patients with AF

**Figure 3.**
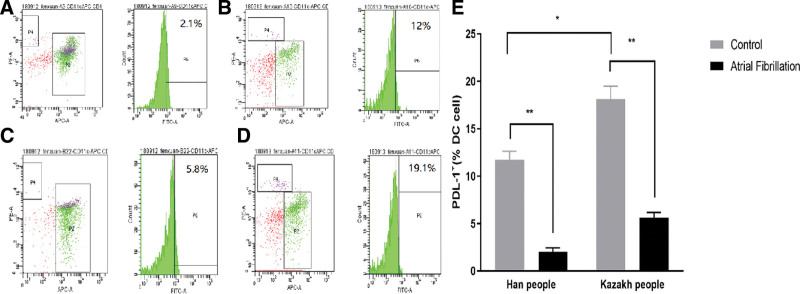
Expression of PD-L1 on the surface of peripheral blood DC cells in the AF and non-AF groups. (A) Han AF; (B) Han non-AF; (C) Kazakhs AF; (D) Kazakhs non-AF; and (E) PD-1 versus PD-L1 is expressed on the surface of the cells; ^*^P < .05, ^**^P < .01. AF = atrial fibrillation, DC = dendritic cell, PD-1 = programmed cell death 1, PD-L1 = programmed death ligand 1.

In the Han population, the positive expression rate of PD-L1 on DCs in the AF group was 3.2 ± 0.46%, while in the control group it was 12.6 ± 0.42%. This difference was statistically significant (*P* < .05), indicating that the expression of PD-L1 on DC cells in the Han Chinese AF population was higher than that in the control group (Fig. 3A, B).

In the Kazakh population, the positive expression rate of PD-L1 on DC cells in the AF group was 4.8 ± 0.47%, compared to 10.7 ± 0.72% in the control group. This difference was also statistically significant (*P* < .05), with the expression of PD-L1 on DC cells in the Kazakh population with AF being higher than that in the control group (Fig. 3C, D).

The flow cytometry results demonstrated that the expression level of PD-L1 on DC cells was significantly lower in the AF groups of both the Han and Kazakh populations compared to the non-AF groups, with this difference being statistically significant (*P* < .01). Further analysis revealed that the expression level of PD-L1 on DC cells was higher in the Kazakh group than in the Han group (Fig. 3E).

### 5.5. Flow cytometry sorted CD4+/CD8+ T cells and DC cells

**Figure 4.**
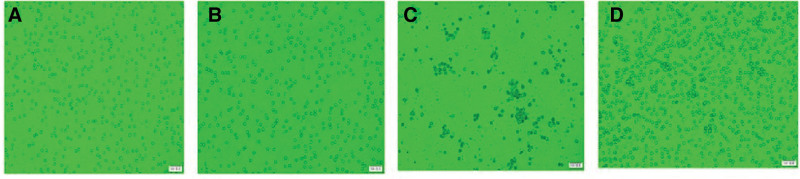
Flow cytometry sorting of cells; (A) CD4+ T cells; (B) CD4+ T cells; (C) DC cells; and (D) DC cells + poly. DC = dendritic cell.

**Figure 5.**
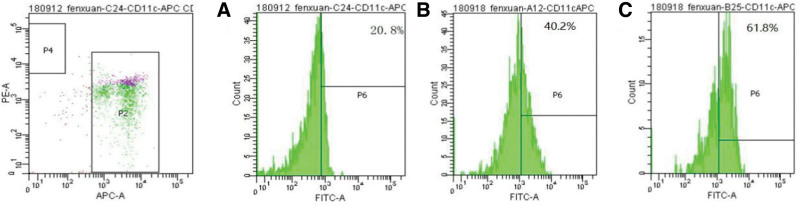
IFN-α interferon up-regulates PD-L1 expression on DC cells; (A) incubated for 0 h; (B) incubated for 24 h; and (C) incubi+ted for 36 h. DC = dendritic cell, IFN-α = interferon-α, PD-L1 = programmed death ligand 1.

After sorting, the purity of isolation exceeded 96%. All observed cells were translucent, round, and demonstrated good cell activity while growing in suspension within the culture medium. These cells were considered suitable for use in the mixed lymphocyte reaction (MLR) experiment. Following the addition of 50 μg/mL poly I:C for 24 hours, the isolated DC cells increased in size and exhibited semi-suspension growth in clusters, further indicating robust cell activity (Fig. 4). Flow cytometry results revealed a significant increase in PD-L1 levels on DC cells over time (Fig. 5).

### 5.6. MLR assay

The number of cells within the isolated immune cells was quantified using flow cytometry. Subsequently, these cells were suspended in DMEM culture medium, and the resulting cell concentration was calculated. The purified DCs and CD4+/CD8+ T cells were introduced into each experimental group at varying ratios. The CD4+ and CD8+ T cells were sourced from the peripheral blood of allogeneic healthy individuals, with the ratios of the 2 cell types established at 1:5, 1:10, 1:20, and 1:40. The cell mixture was cultured in 96-well plates, with a total of 10,000 cells per well and a total volume of 200 μL. The plates were incubated at 37°C in a 5% CO₂ environment for 5 days. Following this incubation period, cell counting kit-8 was added at a concentration of 20 μL per well. After an additional 6 hours of incubation, the plates were analyzed using an enzyme marker at a wavelength of 450 nm to determine their absorbance. A standard curve was plotted to assess cell proliferation. The results demonstrated that:

In the Han population, CD4+/CD8+ T lymphocytes exhibited varying degrees of proliferation in both the AF and control groups following a 5-day mixed culture period, with the highest degree of proliferation observed at a cell ratio of 1:1. The ability of the AF and control groups to stimulate T lymphocyte proliferation revealed discrepancies between the 2 groups, as the AF group demonstrated a superior capacity to promote lymphocyte proliferation compared to the control group. However, this disparity was either eliminated or reversed upon the introduction of IFN-α-treated DCs (Fig. 5A).

**Figure 6.**
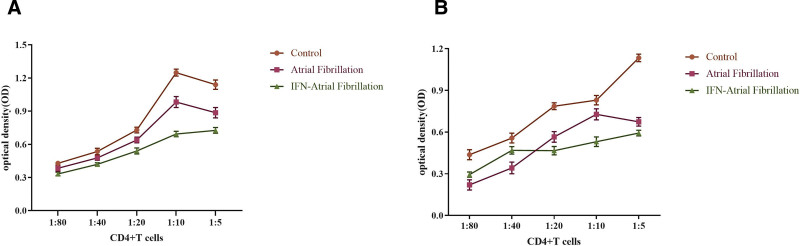
Results of allogeneic MLR experiments in Han (A) and Kazakh (B) patients with and without AF. AF = atrial fibrillation, MLR = mixed lymphocyte reaction.

In the Kazakh population, CD4+/CD8+ T lymphocyte proliferation similarly exhibited varying degrees in both the AF and control groups after the 5-day mixed culture period, with the highest degree of proliferation also noted at a cell ratio of 1:1. The capacity of the AF and control groups to stimulate T lymphocyte proliferation showed discrepancies as well, with DC cells in the AF group displaying a heightened ability to promote lymphocyte proliferation relative to those in the control group. Nonetheless, this distinction diminished or reversed with the introduction of IFN-α-treated DCs (Fig. 6B).

A comparison between the Han and Kazakh populations in Xinjiang revealed that the Han population had a significantly greater capacity to promote lymphocyte proliferation than the Kazakh population (*P* < .05).

### 5.7. Cytokine testing

MLR culture supernatants were collected for subsequent cytokine assays, which tested 7 cytokines: interleukin-2 (IL-2), interleukin-4 (IL-4), IL-6, IL-10, interleukin-17A (IL-17A), tumor necrosis factor (TNF), and interferon-γ (IFN-γ). The standard curves for these cytokines exhibited *R*² values exceeding 0.99.

Significant differences in the secretion levels of IL-2, IL-6, and IL-4 were observed between the Han group and both the AF group and the control group (*P* < .05). Additionally, the culture medium supernatant did not contain detectable levels of IL-17A, TNF, or IFN-γ. The secretion levels of IL-2, IL-6, IL-4, and IL-10 in the IFN-α treatment group were not significantly different from those in the control group (*P* > .05).

In the Kazakh population, the secretion levels of IL-2 and IL-6 differed between the AF group and the control group (*P* < .05). Furthermore, no IL-17A, TNF, or IFN-γ were detected in the culture medium supernatant. The secretion levels of IL-2, IL-6, IL-4, and IL-10 in the IFN-α treatment group also showed no significant differences when compared to the control group (*P* > .05).

**Figure 7.**
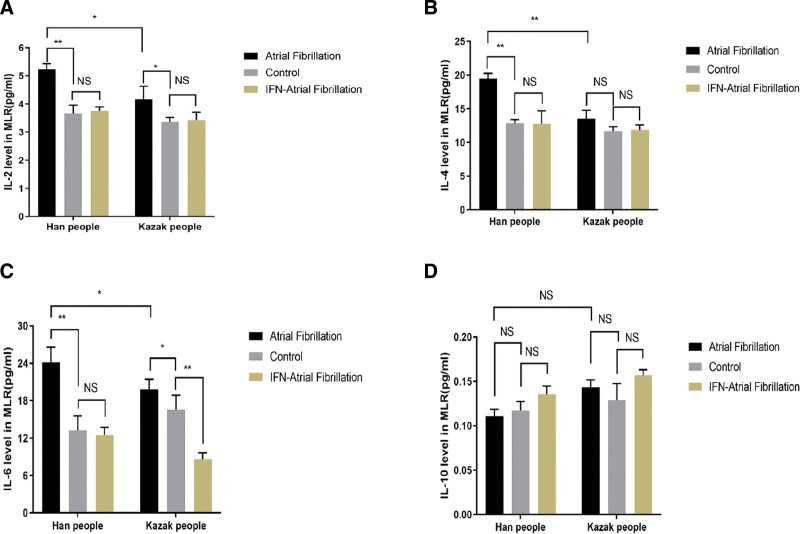
Cytokine levels in the supernatant of the lymphocyte mixing assay; (A) IL-2; (B) IL-4; (C) IL-6; and (D) IL-10; ^*^P < .05, ^**^P < .01. IL-2 = interleukin-2, IL-4 = interleukin-4, IL-6 = interleukin-6, IL-10 = interleukin-10.

Notably, the secretion levels of IL-2, IL-6, IL-4, and IL-10 varied between the Han and Kazakh AF populations (*P* < .05). The Han AF group exhibited higher secretion levels of IL-2, IL-6, and IL-4 compared to the Kazakh AF group, while the IL-10 secretion level was lower in the Han AF group than in the Kazakh AF group (Fig. 7A–D).

## 6. Discussion

### 6.1. Inflammatory response and AF: mechanisms, associations and therapeutic explorations

There is substantial evidence that the inflammatory response plays a significant role in the pathophysiology of AF. This response is associated with various pathological processes in AF, including oxidative stress, apoptosis, and fibrosis, which collectively contribute to the formation of the AF maintenance matrix. Furthermore, inflammation is linked to endothelial dysfunction, platelet activation, and the activation of the coagulation cascade, all of which facilitate thrombosis. Numerous cardiovascular diseases associated with inflammatory responses, such as coronary artery disease, hypertension, heart failure, and cardiomyopathy, have been shown to increase the risk of developing AF, either directly or indirectly. It is plausible that inflammation may lead to the development of AF into an irreversible vicious cycle. Early histological studies have indicated that even in patients with so-called “isolated AF,” localized atrial inflammatory infiltrates are present in 2-thirds of cases, underscoring the role of chronic inflammation in atrial arrhythmias. Additionally, some studies have suggested a link between autoimmune disorders, particularly rheumatoid arthritis, and AF, which may impact atrial electrophysiology. A growing body of evidence indicates that inflammatory mediators can promote atrial remodeling through potential mechanisms such as atrial fibrosis, gap junction regulation, and abnormal intracellular calcium handling. These abnormalities result in increased atrial ectopic electrical activity and a prolonged atrial conduction time. Notably, a recent study involving over 20,000 patients with autoimmune rheumatic diseases demonstrated an association between the inflammatory state, as reflected by CRP levels, and the occurrence of AF.^[16–18]^ It is evident that both systemic and endocardial endothelial inflammation are linked to atrial remodeling. Several inflammatory mediators seem to play a role in the electrical and structural remodeling of the atria. TNF, IL-2, and platelet-derived growth factor regulate calcium homeostasis and contribute to the abnormal triggering of pulmonary veins, as well as the shortening of action potential duration. In conjunction with myeloperoxidase and heat shock proteins, these factors induce atrial fibrosis. Additionally, dysregulation of connexins, apoptosis, and rhabdomyolysis lead to slowed conduction and increased conduction heterogeneity. Furthermore, inflammatory cytokines activate NF-κB (a transcription factor), which contributes to the inflammatory response associated with atrial fibrosis, apoptosis, and cardiomyocyte death. Notably, local inflammatory responses in the atrial myocardium or surrounding tissues may directly induce AF. This mechanism appears to elucidate episodes of AF that occur following pericarditis, myocarditis, and cardiac surgery. It is significant that the peak incidence of postoperative AF occurs on the second and third postoperative days, coinciding with the peak inflammatory and oxidative responses after surgery. Conversely, rapid atrial tachycardia leads to calcium overload-induced oxidative stress, apoptosis, membrane dysfunction, energy depletion, and low-grade inflammation. Furthermore, experimental data indicate that rapid atrial pacing elevates levels of CRP and AF, thereby reinforcing the concept of inflammation-induced AF. Therefore, atrial tachycardia and inflammation are 2 interrelated processes that create a vicious cycle.^[19–22]^ AF may serve as either a cause or a consequence of inflammation. The precise regulatory mechanisms underlying the immune modulation of inflammation in AF remain unclear. Although several inflammatory biomarkers have been associated with an increased risk of AF, their practical utility in guiding AF management has yet to be established. Despite extensive research efforts, no effective anti-inflammatory therapeutic target for AF has been identified. Therefore, it is essential to explore new therapeutic avenues to achieve more effective anti-inflammatory outcomes.

### 6.2. Role of PD-1/PD-L1 in immunotherapy and potential association with AF

PD-1 and its ligand PD-L1 are critical targets for immunotherapy, significantly influencing tumor immunity. Their interaction leads to T-cell dysfunction or even exhaustion, which diminishes the inflammatory response and promotes immune tolerance. This mechanism facilitates conditions that allow tumor cells to evade immune attacks, thereby enabling their survival and proliferation under the “surveillance” of the immune system. As medical research has advanced, high-affinity anti-PD-1 and anti-PD-L1 monoclonal antibodies have been developed. These antibodies are capable of effectively reversing immune tolerance and enhancing the T-cell response. Consequently, the blockade of the PD-1/PD-L1 pathway has been shown to be highly efficacious in the context of anticancer therapy, significantly prolonging the survival of patients with specific tumors. In cases where tumors have developed resistance to conventional chemotherapy, anti-PD-1/PD-L1 agents have been demonstrated to effectively inhibit tumor growth by subtly modulating the interaction between immune cells and tumor cells. To date, 4 checkpoint antibody inhibitors have been employed in immunotargeted therapies targeting PD-1, PD-L1, and CTLA-4. While these immunotherapies have shown efficacy in treating specific cancers, their utility is limited by the fact that their effectiveness is predominantly confined to cancer types with insufficient and heterogeneous expression of PD-1 in the tumor microenvironment. Nevertheless, immunotherapy represents a significant advancement in the field of medicine, with immune checkpoint inhibitors emerging as some of the most successful anticancer therapeutic agents. They have been widely utilized in the treatment of various malignancies, including metastatic melanoma, non-small-cell lung cancer, and other tumors such as breast cancer. The potential for further applications is currently being actively explored.^[23–25]^

In normal physiological conditions, the T cell-mediated immune system is an extraordinarily complex and sophisticated network, comprising numerous stimulatory and inhibitory proteins that collaboratively maintain the balance and stability of immune function. Inhibitory receptors, such as PD-1/PD-L1, play a pivotal role in regulating the activation and function of cytotoxic T lymphocytes, thereby sustaining the body’s intrinsic tolerance and minimizing bystander tissue damage. Upon the binding of T cell receptors to antigens in the presence of major histocompatibility complexes, immune checkpoint molecules modulate the signaling of co-stimulatory factors, such as CD28, which amplifies immune response signals and promotes positive regulation of the immune response. However, PD-1/PD-L1 are expressed on activated T cells and have been shown to inhibit antitumor responses when they interact with ligands on APCs or tumor cells. Consequently, it is essential to shift the balance of the immune microenvironment in favor of antitumor activity and to enhance antitumor effects by targeting and blocking these immunosuppressive interactions through the use of monoclonal antibodies.^[26–28]^ The PD-1/PD-L1 pathway plays a crucial physiological role in regulating the extent of inflammation at the site of antigen expression, thereby safeguarding normal tissue from damage. Notably, PD-1 protein is present on the surface of all activated T cells. Upon recognizing antigens presented by the major histocompatibility complex on target cells, T cells produce inflammatory cytokines, which subsequently drive PD-L1 expression in tissues, activate PD-1 proteins on T cells, and ultimately lead to immune tolerance. This phenomenon is characterized by a loss of control over the inflammatory response, even in the presence of bindable antigens. In certain tumors, such as melanoma, tumor escape is facilitated through the overexpression of PD-L1. Inhibitors of the PD-1/PD-L1 interaction pharmacologically block this interaction, thereby promoting an immune response capable of eliminating tumor cells. Furthermore, PD-1 is expressed on a variety of immune cells, including monocytes, T cells, B cells, DCs, and tumor-infiltrating lymphocytes.^[29–31]^ PD-L1 is expressed in tumor cells and APCs. The interaction between these 2 cell types leads to T-cell dysfunction and promotes the secretion of IL-10, which, in turn, facilitates tumor growth. Furthermore, additional molecular interactions can influence cell activation and the aggressiveness of tumor cells. The blockade of immune checkpoints has been shown to affect the balance between autoimmunity and immune tolerance, potentially resulting in a range of immune-mediated adverse reactions, including fatigue, rashes, dermatological disorders, gastrointestinal events, endocrinopathies, and diarrhea.^[32,33]^ The toxicity associated with anti-PD-1/PD-L1 monoclonal antibodies (mAb) is relatively uncommon and generally less severe than that of anti-CTLA-4 mAb. Nevertheless, treatment with anti-PD-1 and PD-L1 can lead to immune-related adverse events, which may include diarrhea, colitis, pancreatitis, as well as neurological, hematological, and pneumonitis-related complications.^[6]^ Moreover, prolonged treatment may trigger severe immune reactions. Animal studies have shown that PD-1 knockout mice display lymphocyte proliferation, resulting in cardiac enlargement and the accumulation of large inflammatory cell deposits in the enlarged myocardium. Given these observations, it is reasonable to hypothesize that PD-1/PD-L1 may play a critical role in the pathogenesis and persistence of AF. Additionally, the upregulation of PD-1 levels could represent a significant target for modulating the inflammatory response in AF.^[7]^ However, the precise mechanisms by which PD-1/PD-L1 influences immune regulation of inflammation in AF remain to be fully elucidated. Despite a substantial body of evidence supporting the role of inflammation in the pathogenesis and maintenance of AF, there is a notable lack of comprehensive studies on the molecular biological mechanisms underlying inflammatory immunomodulation in this condition. To date, our assessment of the inflammatory burden has primarily concentrated on the secretion of specific cytokines associated with T lymphocytes.

### 6.3. Role of inflammatory biomarkers in AF and related tests

CRP, a protein detectable in serum during the acute phase of inflammation, appears to function more as a marker of latent immunity than as a direct participant in the pathophysiological mechanisms of inflammation. Nonetheless, it can be observed during the inflammatory response. Additionally, various factors influence the level of CRP expression in serum, making CRP a nonspecific inflammatory secretory factor in the inflammatory immune regulation of AF.^[16–18]^ Interleukin-17 (IL-17) is a cytokine secreted by T helper cells 17 (Th17).^[34]^ Th17 cells primarily mediate the differentiation of naïve T cells, a process predominantly driven by IL-6,^[35–37]^ a cytokine primarily secreted by macrophages. IL-6 plays a critical role in neutrophil-infiltrated tissues, inducing apoptosis at the level of T helper cells and polarizing differentiation to favor effector Th17 cells over regulatory T cells.^[38]^ Th17 cells produce IL-17, which is not only responsible for increased fibrosis – a significant factor in the development of AF – but also serves as a cornerstone in the pathophysiology of various diseases, including psoriasis, nonalcoholic fatty liver disease, and metabolic syndrome, thereby contributing to the increased incidence of AF.^[39–41]^ Furthermore, IL-17 is associated with the upregulation of the transforming growth factor β signaling pathway, which is another potent promoter of atrial fibrosis. Additionally, IL-17 stimulates the production of pro-inflammatory cytokines such as TNF-α and IL-6, regulates neutrophil and myocyte apoptosis during tissue infiltration, and can engage in various other pathophysiological pathways, including oxidative stress and a hypercoagulable state.^[42,43]^ Simultaneously, IL-17 induces the production of IL-6, which plays a crucial role in the immune regulation of AF. IL-6 promotes the differentiation of Th17 cells from naive T cells while concurrently inhibiting the regulatory T cells imbalance induced by transforming growth factor β.^[44–46]^ Although inflammatory biomarkers in AF cannot be solely relied upon as standalone evaluation criteria for assessing the inflammatory status of the condition, they do reflect, to some extent, the overall inflammatory burden associated with the disease. Therefore, this section of the study aims to elucidate the inflammatory immune status in patients with AF from the Han and Kazakh populations in the Xinjiang region by examining the proliferative capacity of lymphocytes. This approach will enhance our understanding of the inflammatory processes and their interaction with immune regulation in AF patients of diverse ethnic backgrounds. Such insights will inform subsequent, more comprehensive investigations into the underlying mechanisms of AF and the development of precision-based therapeutic strategies.

### 6.4. Key findings and discoveries of the study

A comparative study of the Han and Kazakh populations in Xinjiang revealed differences in the expression levels of PD-1/PD-L1 on CD4+ T lymphocytes and DCs in the peripheral blood of patients with AF compared to the control group. Notably, the expression of PD-1 on CD4+ T lymphocytes was significantly reduced in both the Han and Kazakh AF groups relative to the non-AF group. Furthermore, the expression level was higher in the Kazakh group compared to the Han population. In addition, the expression of PD-L1 on DC cells was significantly lower in both the Han and Kazakh AF groups than in the non-AF group, with the Kazakh group exhibiting higher levels than the Han group. Studies have also shown that Kazakh patients with AF demonstrate a reduced inflammatory burden compared to Han Chinese individuals, which may be linked to genetic variations, lifestyle factors, and environmental influences. Moreover, there are significant differences in cytokine secretion levels between the Han and Kazakh AF populations. Specifically, the levels of IL-2, IL-6, and IL-4 secretion are elevated in the Han AF group compared to the Kazakh AF group, while IL-10 secretion is lower in the Han AF group than in the Kazakh AF group. These findings are consistent with the expression levels of PD-1/PD-L1 and T cell proliferation, further supporting the idea that PD-1/PD-L1 expression plays a crucial role in the immune regulation of AF.

### 6.5. Analysis and discussion of results

The PD-1/PD-L1 pathway plays a pivotal role in immunomodulation, regulating the immune system through intricate mechanisms and significantly influencing the maintenance of immune homeostasis. In patients with AF, the expression levels of PD-1/PD-L1 on CD4+ T lymphocytes and DCs in the peripheral blood have been found to be reduced to varying degrees, suggesting that this pathway may be involved in the pathogenesis of AF. A reduction in PD-1/PD-L1 expression disrupts the equilibrium of immune regulation, leading to the overactivation of immune cells and the release of numerous pro-inflammatory factors, including TNF-α and IL-6. These factors have deleterious effects on cardiac tissues. An extended inflammatory response can result in pathological alterations, including damage and fibrosis of cardiomyocytes, which disrupt the normal structure and function of the heart, contributing to the development of AF.

The results of the study indicated that the burden of certain inflammatory indicators was lower in Kazakh AF patients compared to their Han Chinese counterparts. Several factors may contribute to this difference. Genetically, variations in the genetic composition between ethnic groups can influence the expression and regulation of immune-related genes. Specific gene variants may enable Kazakhs to modulate their inflammatory response differently than Han Chinese individuals, who generally exhibit a milder inflammatory response. Lifestyle factors also play a significant role; the 2 ethnic groups differ in terms of diet, exercise, and daily routines. The traditional Kazakh diet may include specific nutrients, such as antioxidants, that help modulate the inflammatory response. Additionally, environmental factors should not be overlooked. Kazakhs typically reside in grassland and mountainous regions characterized by clean air and low pollution levels, which positively impact the immune system and help reduce inflammation. In contrast, the Han Chinese population is widely dispersed, with some areas experiencing poorer environmental conditions that may stimulate the immune system and elevate inflammatory burdens. A comprehensive examination of these differences is essential for understanding the pathogenesis of AF. Analyzing genetic, lifestyle, and environmental factors across different ethnic groups may elucidate the mechanisms linking inflammation and AF. Furthermore, investigating the relationship between genetic variants and inflammatory responses could identify potential targets for genetic diagnosis and treatment of AF. Understanding the influence of lifestyle on AF may also lead to tailored prevention strategies. Moreover, Kazakhs exhibit higher levels of PD-1/PD-L1 expression on CD4+ T lymphocytes, CD8+ T lymphocytes, and DCs compared to Han Chinese individuals. This observation suggests potential differences in immunoregulatory mechanisms between the ethnic groups that may influence susceptibility to AF and therapeutic responses. Further studies are warranted to explore the clinical significance of this finding.

The findings of this study identify a novel intervention target for the treatment and management of AF patients of Han and Kazakh ethnicity in Xinjiang. This approach may enhance the immune status of AF patients, attenuate the inflammatory response, and improve therapeutic efficacy by modulating the PD-1/PD-L1 pathway. Further research could elucidate the precise mechanisms of the PD-1/PD-L1 pathway in the etiology of AF and the biological basis for the observed differences between ethnic groups. Larger clinical studies should be conducted to verify the reliability of these results and to investigate the potential efficacy of personalized treatment based on the PD-1/PD-L1 pathway in patients with AF.

## 7. Conclusion

The present study conducts an in-depth analysis of immune cell profiles in patients with AF from the Han and Kazakh populations in Xinjiang, revealing a strong link between AF and the immune system. The reduced expression levels of PD-1 and PD-L1 in CD4+ T cells, CD8+ T cells, and DCs in patients with AF lead to the overactivation of T lymphocytes, which increases the inflammatory load. This, in turn, promotes cardiomyocyte fibrosis and structural remodeling, playing a key role in the pathogenesis of AF. Contrary to the conventional view that inhibitors are employed to reduce PD-1/PD-L1 pathway levels to enhance immunity, the present study demonstrates that elevated levels of PD-1/PD-L1 expression hold positive significance in the immune regulation of AF. This regulation can reduce the inflammatory load and influence the mechanisms underlying the development and maintenance of AF. These findings provide a novel target and direction for anti-inflammatory treatment of AF. Further investigation into the specific role of the PD-1/PD-L1 pathway in the pathogenesis of AF is recommended to establish a more solid theoretical basis and develop more effective therapeutic strategies for clinical treatment. It is anticipated that continued research and development will yield more effective strategies for managing AF, a prevalent cardiovascular disorder, thereby enhancing the quality of life and well-being of patients while advancing the prevention and treatment of cardiovascular diseases.

## Acknowledgments

We would like to express our sincere gratitude to the First Affiliated Hospital of Xinjiang Medical University for their support in conducting this research and providing access to the patient population from the Department of Comprehensive Cardiology. We also thank the participants of the study from both the Han and Kazakh populations for their invaluable contributions.

## Author contributions

**Conceptualization**: Wen Bai, Muhuyati Wulasihan.

**Data curation**: Wen Bai, Mierzhati Maimaiti, Muhuyati Wulasihan.

**Formal analysis**: Wen Bai, Mierzhati Maimaiti.

**Funding acquisition**: Muhuyati Wulasihan.

**Investigation**: Wen Bai, Mierzhati Maimaiti, Xinwen Zhang, Muredili Saimi, Muhuyati Wulasihan.

**Methodology**: Xinwen Zhang, Xia Zhu.

**Project administration**: Wen Bai, Muhuyati Wulasihan.

**Resources**: Xinwen Zhang, Xia Zhu, Muredili Saimi.

**Software**: Wen Bai, Mierzhati Maimaiti, Xinwen Zhang, Muredili Saimi.

**Supervision**: Mierzhati Maimaiti, Xinwen Zhang, Xia Zhu, Muredili Saimi.

**Validation**: Mierzhati Maimaiti, Xia Zhu, Muredili Saimi.

**Visualization**: Xia Zhu, Muredili Saimi.

**Writing** – **original draft**: Wen Bai.

**Writing** – **review & editing**: Muhuyati Wulasihan.
